# Reduced but Reversible Brain Entropy After Occupational Partial Sleep Deprivation in Night‐Shift Medical Staff

**DOI:** 10.1002/brb3.71530

**Published:** 2026-06-19

**Authors:** Siqi Cai, Fan Yang, Chunxiang Jiang, Lixian Zou, Lijuan Zhang

**Affiliations:** ^1^ Paul. C. Lauterbur Research Centers for Biomedical Imaging, State Key Laboratory of Biomedical Imaging Science and System, Shenzhen Institutes of Advanced Technology Chinese Academy of Sciences Shenzhen China; ^2^ University of the Chinese Academy of Sciences Beijing China; ^3^ Key Laboratory of Biomedical Imaging Science and System Chinese Academy of Sciences Shenzhen China

**Keywords:** brain entropy, night‐shift work, psychomotor vigilance, and sleep deprivation

## Abstract

**Background:**

Partial sleep deprivation (PSD) poses health risks to the night‐shift workers (NSW), but its underlying impacts on local brain function remain underexplored. Brain entropy (BEN), a nonlinear dynamic metric, has emerged as a novel parameter of choice in probing the temporal irregularity of brain activity, thus may offer new insights into the characterization of the brain dysfunction following sleep deprivation.

**Methods:**

Seventy‐eight female medical NSWs and 30 non‐NSW healthy controls (HC) were recruited in this study. Psychomotor vigilance tasks (PVT) and resting‐state fMRI (rs‐fMRI) were sequentially conducted at three conditions for NSWs: baseline (prior to NSW), PSD (immediately after NSW), and recovery (after 3–5 days of regular sleep). Nine NSWs were excluded due to consecutive or intermittent sleep duration exceeding 6 h on the night‐shift day, or significant head motion in the rs‐fMRI scans, resulting in 69 NSWs in the following analysis. Static and dynamic sample entropy (SampEn) metrics were calculated for BEN quantification. An L1‐regularized logistic regression (LR) model was constructed based on baseline SampEn metrics to distinguish PSD‐vulnerable (*N* = 35) and PSD‐resistant (*N* = 34) individuals with their PVT performances as the classification reference.

**Results:**

Compared to HCs, SampEn of young female NSWs reduced mainly in the occipital and temporal cortices, and associated with poor sleep quality. But these inter‐group differences did not survive the strict multiple comparison correction. Both static and dynamic SampEn metrics of NSWs were significantly altered following acute PSD, and largely returned to the baseline after sleep recovery. The SampEn‐based LR model achieved a classification accuracy value of 78.26% to distinguish PSD‐vulnerable and PSD‐resistant individuals.

**Conclusions:**

One‐night of acute PSD in shift work leads to reduced but largely reversible BEN, whereas long‐term occupational chronic PSD tends to reduce the BEN in distributed primary cortices and correlates with the poor sleep quality in young female NSWs. The BEN metrics could serve as a potential biomarker to identify individual susceptibility to PSD, which may contribute to the optimization of rotating‐shift schedules.

## Graphical abstract

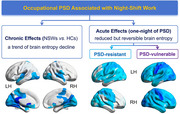

## 1 Introduction

Sleep deprivation has become increasingly prevalent in modern society. Long‐term exposure to sleep deprivation may disrupt the circadian rhythms and sleep homeostasis (Boivin et al. 2022; Yook et al. 2024; Nilsson et al. 2025), and increase the risks of cognitive impairment (Marquié et al. 2015), emotional disruption (Jørgensen et al. 2021), and cerebrovascular events (Gottesman et al. 2024). Medical staff engaging in night‐shift work (NSW) represents a classic occupational population experiencing chronic partial sleep deprivation (PSD). The immediate drawbacks following PSD in night‐shift work are mental and physiological fatigue and vigilance decline, which could impair the job performance of medical workers and increase the probability of medical errors (Behrens et al. 2019; Di Muzio et al. 2019). Clarifying the impact of PSD on their brain function is crucial not only for the healthiness of the shift‐work individuals, but also for the excellence of medical services. Recent studies have confirmed that both acute and chronic sleep deprivation remodel the functional profile of the brain in terms of the static and dynamic functional connectivity in distributed brain regions (Chen et al. 2018; Xu et al. 2018; Chen et al. 2022). The altered functional synchronization was found to be closely associated with the sustained attentional impairments and working memory decline after sleep deprivation (Cai et al. 2021; Feng et al. 2023). Moreover, these cognitive and neurobehavioral vulnerabilities exist significant individual differences, which are stable and trait‐like (Yamazaki and Goel 2020). Identifying the individual vulnerability to sleep deprivation is crucial for optimizing rotating‐shift scheduling, and thereby mitigating the health risks associated with night work (Waage et al. 2021).

However, in contrast to the extensive evidence of the changes in inter‐regional interaction and functional integration, the response of local brain activity to sleep deprivation remains underexplored. Since the temporal neuronal coherence is fundamental to communication across regions (Fries 2015), the investigation of PSD's impacts on brain function would benefit from assessing the fluctuating activity of local neuronal ensembles. As a nonlinear dynamic metric, brain entropy (BEN) has emerged as a novel parameter for probing the temporal irregularity and complexity of brain spontaneous fluctuations (Wang 2021). High BEN reflects a greater diversity of neural activity patterns, thus characterizing the brain's capacity to flexibly reconfigure neural resources in response to changing cognitive demands (Del Mauro and Wang 2024). Emerging evidence has shown that BEN metrics were highly sensitive to brain diseases (Xue et al. 2019; Wang and Alzheimer's Disease Neuroimaging Initiative 2020), sleep/wake states (Bruce et al. 2009; Yang et al. 2024) and mental fatigue (Peng et al. 2021). These findings collectively indicate that BEN captures physiologically meaningful variations in neural flexibility and information processing capacity, and may provide a new avenue to characterize the functional alterations of the brain following PSD.

Previous studies were mainly conducted under controlled laboratory conditions, which may be insufficient to simulate the real‐world PSD situations in high‐workload night‐shifts. Therefore, we recruited night‐shift medical workers in the present study, aiming to estimate the impact of acute PSD on brain function based on resting‐state functional MRI (rs‐fMRI) and BEN analysis at three time points: prior to NSW, immediately after a sleep‐restricted night‐shift, and after several days of regular sleep. In addition, the inter‐group comparison of BEN was performed between night‐shift workers and healthy controls at the baseline condition with one night of good sleep in order to investigate the effect of chronic PSD. Furthermore, the night‐shift participants were classified into subgroups of PSD‐vulnerable and PSD‐resistant based on their performance during the psychomotor vigilance task (PVT). Finally, an L1‐regularized logistic regression (LR) model was introduced to assess the utility of brain entropy in predicting the susceptibility to sleep deprivation at the individual level.

## 2 Participants and Methods

### 2.1 Participants

This study was approved by the local institutional review board (registration number: SIAT‐IRB‐240115‐H0711). Participants provided written informed consent before taking part in the experiments. A total of 92 medical staffs (78 females) with regularly scheduled NSW were consecutively recruited from the local hospitals from June 2024 to September 2025. Given that the majority of the participants were female—consistent with the gender composition of the local nurse population (Lu et al. 2021), we included female NSWs only in the following analysis to eliminate gender‐related confounds (*n* = 78, aged 26.15 ± 4.17 years old). Inclusion criteria were rotated through a regular night‐shift calendar for at least 1 month, no history of sleep disorders, neurological and psychiatric diseases, and no history of neuropsychiatric or sleep‐altering medications in the past month. In addition, 30 age‐matched non‐NSW healthy female subjects (aged 25.27 ± 3.85 years old) were recruited as the healthy controls (HC) according to the following inclusion criteria: no history of sleep disorders or neurological and psychiatric diseases, no history of neuropsychiatric or sleep‐altering medications in the past month, no cross‐meridian travel, night‐shift work routines within 6 months before the experiment; normal sleep patterns.

### 2.2 Study Procedure

**FIGURE 1:**
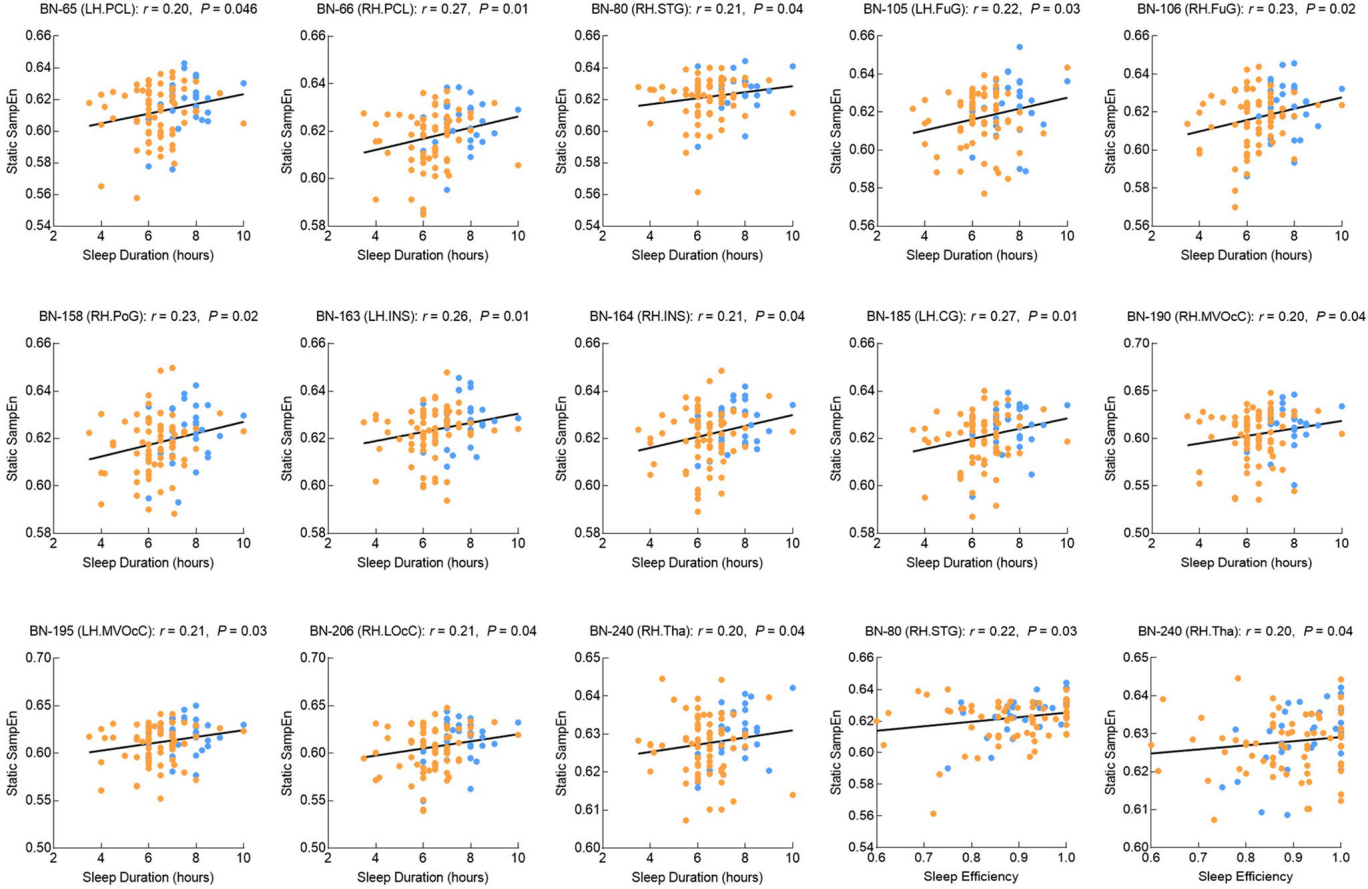
Illustration of the study procedure. BS, baseline condition; HC, healthy control; PSD, partial sleep deprivation; PSD‐Res, resistant to partial sleep deprivation; PSD‐Vul, vulnerable partial sleep deprivation; Rec, sleep recovery condition; SampEn, sample entropy; LR, logistic regression.

The study design was illustrated in Figure 1. For each night‐shift participant, an MRI scan and PVT test were successively conducted in the lab at three wakefulness conditions: (1) baseline wakefulness condition (BS, prior to a sleep‐restricted NSW), (2) PSD condition (PSD, immediately after NSW), and (3) three to five days after NSW with sleep recovery condition (Rec). Participants were required to maintain a regular sleep‐wake schedule prior to the baseline session. The normal sleep was verified by their self‐reports and the objective recording of personal consumer‐grade wrist‐worn activity trackers, failing which, the study would be rescheduled. Additionally, the night‐shift participants with a total sleep duration over 6 h during the 24‐h NSW day would be excluded from the subsequent analyses.

### 2.3 Behavioral Assessment

The subjective sleep quality of participants was assessed using the global Pittsburgh Sleep Quality Index (PSQI) and the Athens Insomnia Scale (AIS) questionnaires. The self‐report depression scale was assessed using Beck Depression Inventory‐II (BDI‐II). In addition, a 10‐min visual PVT test was performed right before the MRI scan to estimate the mental fatigue and alertness level of participants (Reifman et al. 2018). The PVT test is highly reproducible and widely validated for vigilance assessment (Drummond et al. 2005). During the PVT task in this study, the participant was instructed to monitor a computer screen (ThinkPad P51S, screen resolution 1920 × 1080 Hz) and pressed a mouse button as quickly as possible upon the appearance of a target stimulus, with the reaction time provided as response feedback. The PVT protocol is repeated for 10 min with varied inter‐stimulus intervals (randomly ranging from 2 s to 10 s). Trials with reaction times longer than 500 ms were defined as minor lapses. The differences in the count of the minor lapse among the three experiment states were calculated and defined as $\Delta_{PSD-BS}$, $\Delta_{PSD-Rec}$, and $\Delta_{Rec-BS}$, based on which the NSW participants were categorized into two subgroups (C. Wang et al. 2022; Yamazaki et al. 2022): the PSD‐resistant subgroup (PSD‐Res, both the $\Delta_{PSD-BS}$ and $\Delta_{PSD-Rec}$ smaller than or equal to two) and the PSD‐vulnerable subgroup (PSD‐Vul, either $\Delta_{PSD-BS}$ or $\Delta_{PSD-Rec}$ exceeding two).

### 2.4 MRI Protocol

All MRI data was acquired on a 3.0T MRI scanner (uMR 790, United Imaging Healthcare, Shanghai, China) equipped with a 32‐channel phased array head coil. Resting‐state fMRI images were collected using a simultaneous multi‐slice (SMS) echo‐planar imaging (EPI) sequence with a repetition time (TR) of 1000 ms, an echo time of (TE) 30 ms, a multi‐band factor of 4, a flip angle (FA) 62°, field of view (FOV) of 210 × 210 mm^2^, an acquisition matrix of 84 × 84, 60 slices with a thickness of 2.5 mm, and 540 volumes. A high‐resolution T1‐weighted structural image was acquired using the fast spoiled gradient echo imaging sequence (GRE‐FSP): TR/TE 8.6/3.3 ms, FA 10°, FOV 243 × 243 mm^2^, acquisition matrix 304 × 304, voxel size 0.8 × 0.8 × 0.8 mm^3^. During MRI data collection, foam cushions were applied to reduce the involuntary head movement.

### 2.5 MR Image Preprocessing

Rs‐fMRI data was preprocessed using the DPABI toolbox (Yan et al. 2016) with the following steps: removal of the first 10 volumes, slice timing, realignment for head motion correction, spatial normalization to the standard Montreal Neurological Institute (MNI) space and smoothing with a 4‐mm full‐width half‐maximum kernel (FWHM), regression of nuisance variables including the mean signal of white matter and CSF as well as Friston's head motion parameters, linear trend removal, and band‐pass filtering (0.01–0.1 Hz). The participants with excessive head motion (translation over 3.0 mm or rotation in any direction over 3.0°) in the rs‐fMRI scan of any study condition were excluded.

### 2.6 Calculation of Static Sample Entropy

Sample entropy (SampEn) was applied to characterize the temporal irregularity of the rs‐fMRI time series (Richman and Moorman 2000). A tolerance factor *r* is introduced as a threshold to assess the inter‐segment match. The pattern length (*m*) and tolerance factor (*r*) of SampEn were optimally set to be 2 and 0.24, respectively, for the Z‐normalized BOLD signals in the present study according to a SampEn parameter selection strategy proposed in previous studies (Figure S1) (Yang et al. 2018; Xin et al. 2024). The voxelwise SampEn map was generated for each participant. Subsequently, the region‐level SampEn value was extracted and averaged for each region of interest (ROI) of the Brainnetome atlas (Fan et al. 2016) for the following analyses.

### 2.7 Temporal Properties of Dynamic Sample Entropy

The sliding‐window approach with a Hamming window of 100 TRs and a sliding step of 6 TRs was introduced to estimate the SampEn dynamics, resulting in a 246 (number of ROIs) × 72 (number of windows) dynamic SampEn (dynSampEn) matrix for each rs‐fMRI session.

In addition, *K*‐means clustering analysis was utilized to identify reoccurring SampEn states across all dynSampEn vectors, using the L1 distance as the measure of similarity. The *K*‐means algorithm was repeated 100 times to minimize the bias induced by initial random selection of cluster centroids. The optimal cluster number was selected by evaluating the additional variance explained as *K* increased (Figure S2). Finally, the fractional window (FW, the occurrence proportion of each state), mean dwell time (MDT, the average number of consecutive windows assigned to one state), the state transition probability, and total transition number were calculated to characterize the temporal properties of brain entropy dynamics.

### 2.8 Classification of PSD‐Res and PSD‐Vul Subjects

An L1‐regularized logistic regression (LR) model was constructed to assess the predictive ability of SampEn features in distinguishing PSD‐Vul and PSD‐Res participants (Figure S3). A leave‐one‐out cross‐validation (LOOCV) framework was applied to separate the night‐shift participants into train and test datasets. In each LOOCV iteration, the principal component analysis (PCA) was applied to the training set using pre‐selected candidate SampEn features that showed inter‐subgroup difference (PSD‐Vul vs. PSD‐Res, two‐sample *t*‐test, *p* < 0.05). The first components accounting for 85% of the cumulative variance were retained as predictive features. The LR model was then trained on the dimension‐reduced training set, with the regularization parameter (λ) optimized via inner 5‐fold cross‐validation. The optimized model was subsequently used to predict the held‐out test sample. Furthermore, the statistical significance of the classification performance was evaluated via a permutation test with the raw labels of subjects randomly shuffled 1000 times.

### 2.9 Statistical Analysis

The group difference in age and mean reaction time (meanRT) of PVT tests was estimated using the two sample *t*‐test (two‐tailed). The group difference in PSQI, AIS, and BDI‐II scores as well as the minor lapse counts of PVT tests were compared with the Mann‐Whitney U test. A Chi‐square test was utilized to evaluate the group difference in the information of night‐shift work, including the occupation (doctor or nurse), napping on the night‐shift work (yes or no), work months of a regular night‐shift schedule (shorter or longer than 6 months), NSW pattern (12 h or 24 h) and NSW frequency (≤ 1 time a week or ≥ 2 times a week). Furthermore, the meanRT and the minor lapse counts were compared among three conditions (BS, PSD, and Rec) using one‐way repeated measures ANOVA, followed by Tukey's multiple comparison test for post hoc pairwise comparisons. The difference in static and dynamic SampEn features among three conditions was estimated using one‐way repeated measures analysis of covariance (ANCOVA), followed by Tukey's multiple comparison test for post hoc pairwise comparisons. The mean framewise displacement (FD) parameters were set as covariance. The inter‐group difference in static and dynamic SampEn features was estimated using a two sample *t*‐test (two‐tailed) with age and mean FD as covariances. The significance level was set at *p* < 0.05 with false discovery rate (FDR) correction.

## 3 Results

### 3.1 Demographics and Subjective Sleep Quality

One night‐shift participant was excluded from the study due to their consecutive or intermittent sleep duration exceeding 6 h on the NSW day, and eight night‐shift participants were excluded due to the significant head motion in the rs‐fMRI scans, especially at the PSD condition, resulting in 69 NSW participants in the following analysis. The mean FD of NSWs after PSD (0.08 ± 0.03) was significantly higher than the baseline condition (0.07 ± 0.03) (*p* < 0.05) (Figure S4), suggesting a PSD‐induced involuntary head motion. There was no significant difference between NSWs and HCs in age, head motion, or the PVT performance (all *p* > 0.05) (Table 1). The BDI‐II, AIS, and PSQI total scores of the NSW group were significantly higher than those of HCs (BDI‐II, *p* < 0.05; AIS, *p* < 0.001; PSQI, *p* < 0.001). In addition, the seven sub‐item scores of PSQI were further compared between HCs and NSWs, with NSWs showing significantly higher scores in sleep latency, sleep duration, sleep disturbance, and daytime dysfunction, suggesting a poorer sleep quality (Table S1).

**TABLE 1:** Demographics, sleep questionnaire, and PVT measures of healthy controls and night‐shift‐work participants.

Groups of Night‐Shift Work (NSW) Participants And Healthy Controls (HC)					
Characteristic		NSW (*n* = 69)	HC (*n* = 30)	Statistical Analysis	
				*t/Z*	*P*
Age (years)^a^		26.22 ± 4.28	25.27 ± 3.85	1.05	0.30
BDI‐II score^b^		8.00 (2.00, 11.00)	4.00 (2.00, 8.00)	−1.96	0.049*
AIS score ^b^		6.00 (3.00, 8.00)	3.50 (1.75, 5.00)	−3.61	<0.001^*^
PSQI score ^b^		6.00 (5.00, 9.00)	3.00 (2.00, 5.00)	−4.80	<0.001^*^
PVT Test	Mean Reaction Time (ms) ^a^	304.44 ± 34.76	313.58 ± 42.87	−1.12	0.27
	Minor Laps Counts (reaction < 500 ms) ^b^	2.00 (1.00, 3.00)	2.50 (1.00, 4.00)	−1.24	0.22

Groups of Resistant And Vulnerable to Partial Sleep Deprivation in Night‐Shift Work						
Characteristic			PSD‐Res (*n* = 34)	PSD‐Vul (*n* = 35)	Statistical Analysis	
					*t/Z*	*P*
Age (years) ^a^			26.56 ± 5.51	25.89 ± 2.64	0.65	0.52
BDI score ^b^			6.00 (3.00, 11.00)	8.00 (1.00, 11.00)	−0.02	0.98
AIS score ^b^			7.00 (3.00, 8.00)	6.00 (4.00, 8.00)	−0.22	0.83
PSQI score ^cb^			7.00 (5.00, 9.00)	6.00 (4.00, 9.00)	−0.46	0.65
PVT Test	Mean Reaction Time (ms) ^a^	BS	297.15 ± 33.07	311.52 ± 35.36	−1.74	0.09
		PSD	303.30 ± 26.62	386.45 ± 111.13	−4.25	<0.001*
		Rec	292.09 ± 33.30	309.86 ± 35.59	−2.14	0.04*
	Minor Laps Counts (reaction < 500 ms) ^b^	BS	1.00 (0.00, 2.00)	2.00 (1.00, 5.00)	−2.55	0.01*
		PSD	2.00(1.00, 2.00)	9.00 (6.00, 13.00)	−6.85	<0.001*
		Rec	1.00 (0.00, 3.00)	2.00 (0.00, 4.00)	−1.30	0.19

Note: ^a^ represents the two‐sample *t*‐test (two‐tailed) with data expressed as mean ± standard deviation. ^b^ represents the Mann‐Whitney U test with data expressed as median (interquartile range). * represents significant intergroup difference with *P* < 0.05.

### 3.2 Reduced Static SampEn Associated With Chronic PSD

**FIGURE 2:**
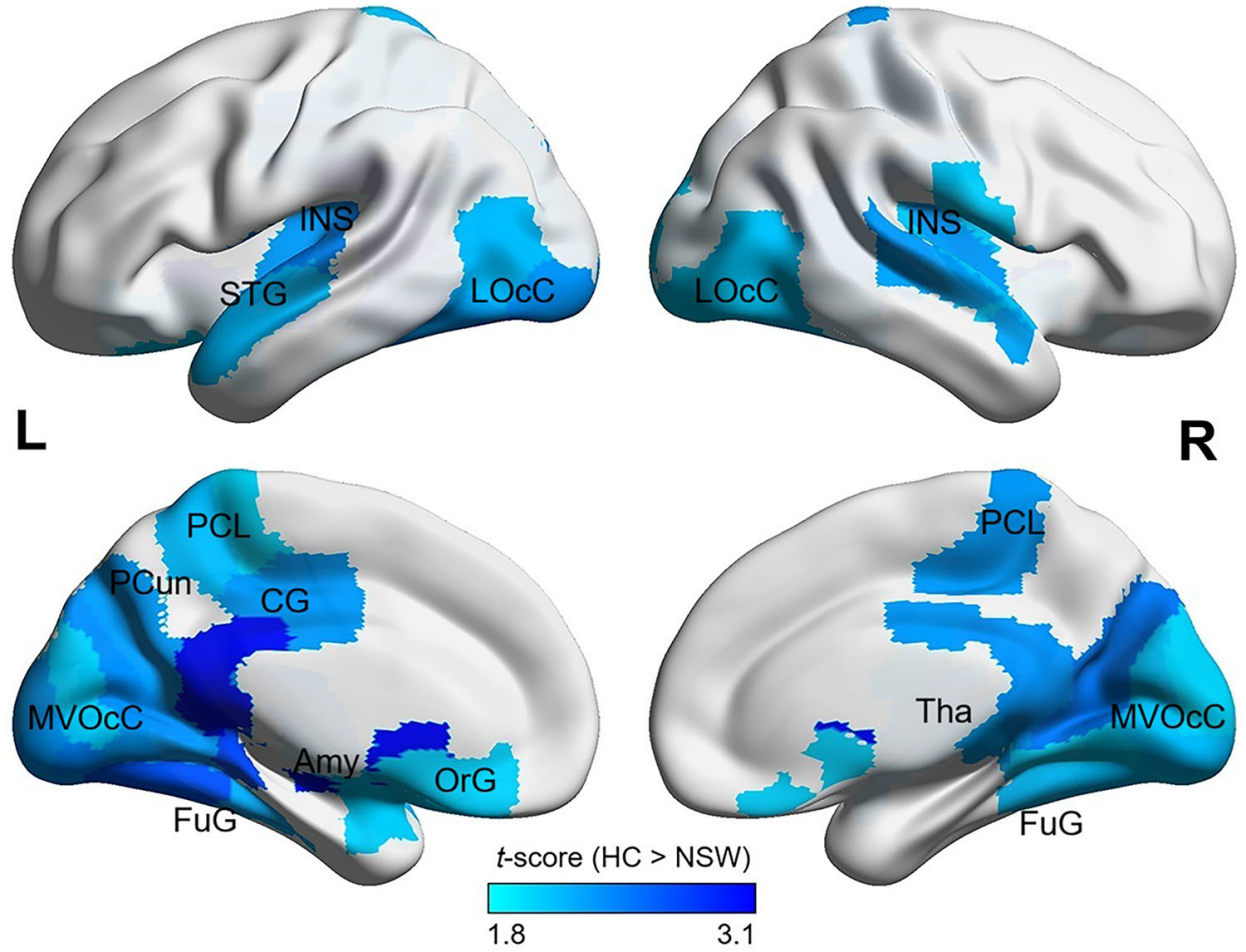
Reduced brain sample entropy associated with chronic partial sleep deprivation. Young female night‐shift workers showed lowered static SampEn mainly involving the occipital and temporal cortices, as compared with HCs (Two‐sample *t*‐test, *p* < 0.05, without FDR correction). L, left hemisphere; R, right hemisphere; OrG, orbital gyrus; PCL, paracentral lobule; Pcun, precuneus; STG, superior temporal gyrus; FuG, fusiform gyrus; INS, insular; CG, cingulate gyrus; MVOcC, medioventral occipital cortex; LOcC, lateral occipital cortex; Amy, amygdala; Tha, thalamus.

The group‐averaged static SampEn maps were shown in the supplemental materials (Figure S5). Compared to HCs, NSW participants showed lower SampEn within 37 ROIs of the Brainnetome atlas under the baseline condition, mainly involving the occipital cortex, superior temporal cortex (STG), fusiform gyrus (FuG), and partial subcortical nuclei (Figure 2), suggesting a decreased temporal irregularity of BOLD signals. However, these inter‐group differences did not survive the strict FDR multiple correction (Figure S6).

**FIGURE 3:**
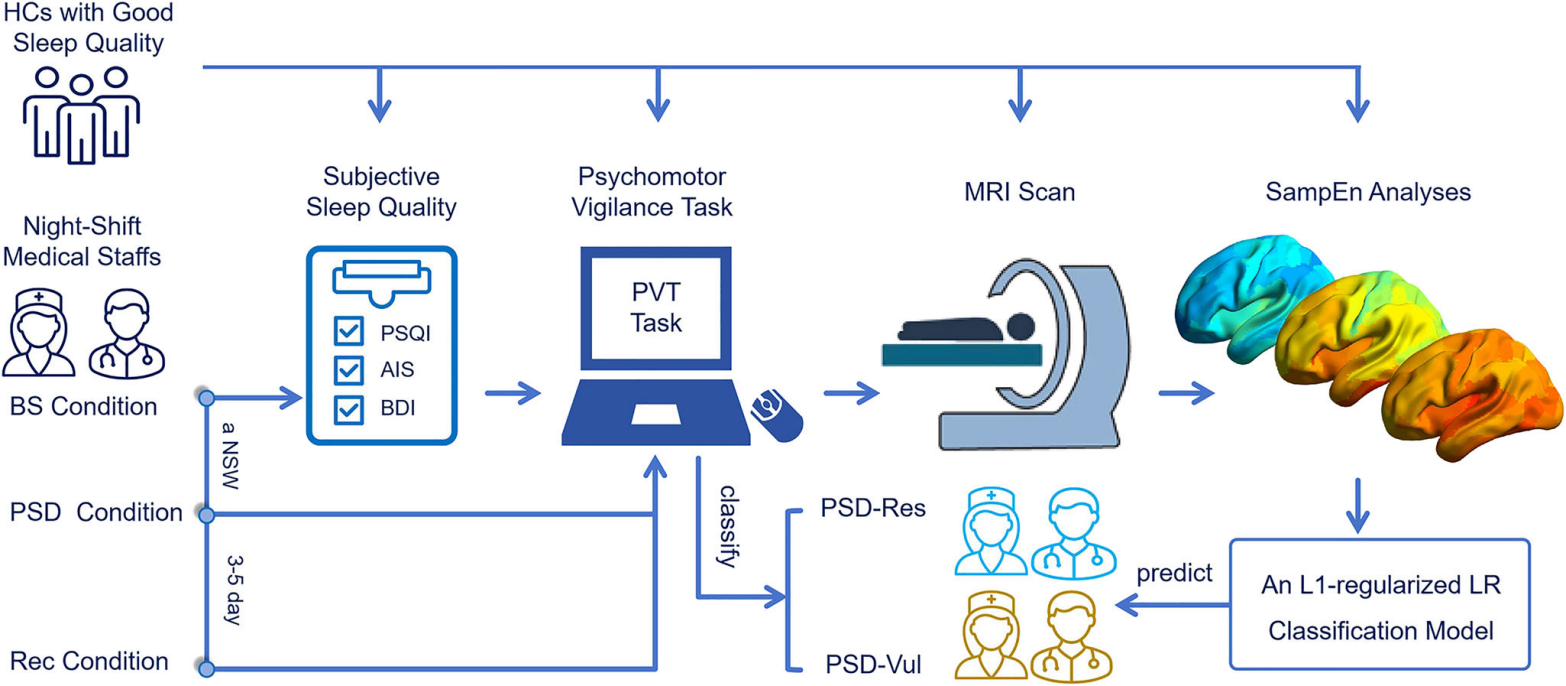
Sensitive brain regions with the SampEn metrics correlated with sleep duration and efficiency. The orange and blue dots represent night‐shift and healthy participants, respectively. BN, Brainnetome atlas; LH, left hemisphere; RH, right hemisphere; PCL, paracentral lobule; STG, superior temporal gyrus; FuG, fusiform gyrus; PoG, postcentral gyrus; INS, insular; CG, cingulate gyrus; MVOcC, medioventral occipital cortex; LOcC, lateral occipital cortex; Tha, thalamus.

The Spearman correlation analyses were further performed to estimate the association between these sensitive SampEn features and the subjective sleep quality scores, including the AIS, PSQI total, and sub‐item scores. A significant positive correlation was identified between the sleep duration and the SampEn features in sub‐regions of the paracentral lobule (PCL, BN‐65, and BN‐66), postcentral gyrus (PoG, BN‐158), STG (BN‐80), FuG (BN‐105 and BN‐106), insular (BN‐163 and BN‐164), caudal area of the cingulate gyrus (CG, BN‐185), medioventral (MVOcC, BN‐190, and BN‐195) and lateral occipital cortices (LOcC, BN‐206), and posterior parietal thalamus (Tha, BN‐240), and between the sleep efficiency and SampEn of BN‐80 and BN‐240 (Figure 3). These sensitive brain areas were mainly located within the somatosensory and motor network (SMN) and the visual network (VN). Taken together, the brain activity of the chronic PSD subjects becomes less flexible following a period of night‐shift work, with alterations predominantly involving the occipital and temporal cortices, and significantly correlated with poor sleep quality.

### 3.3 Effects of Chronic and Acute PSD on Dynamic SampEn Features

**FIGURE 4:**
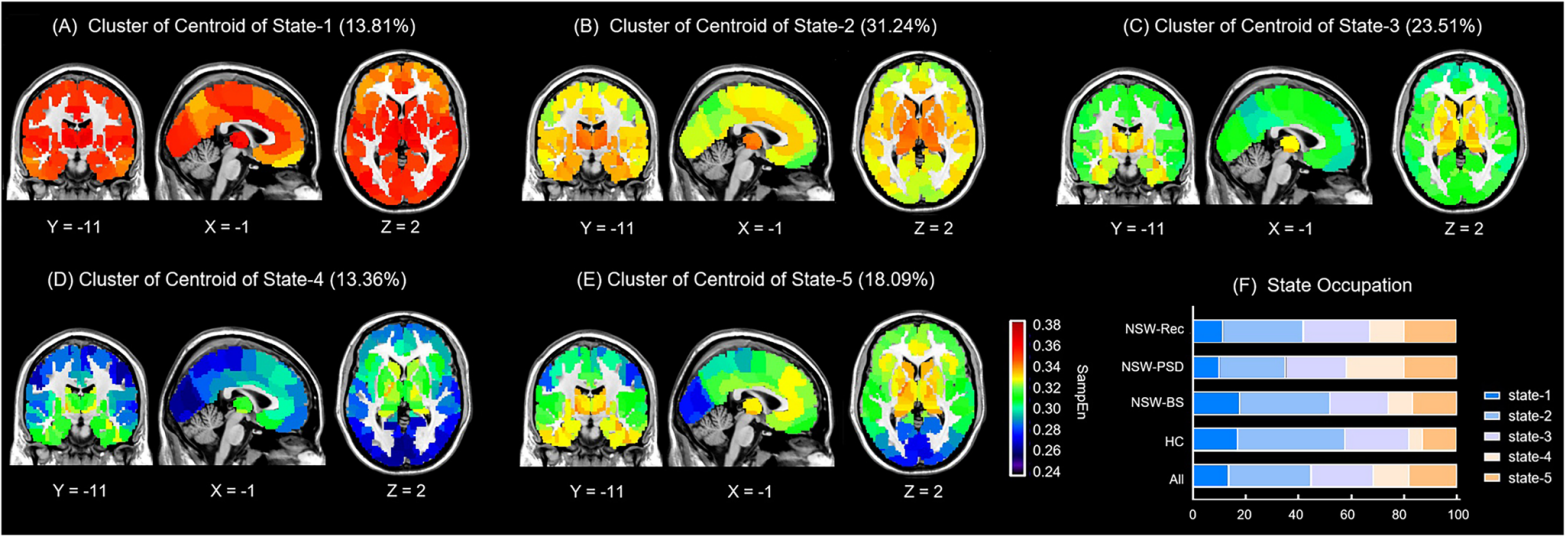
Different and recurrent brain states. (A–E) The cluster centroids of five brain states, respectively. (F) The occurrence frequency of brain states for all participants, healthy controls (HC), and night‐shift workers (NSW) at three different conditions. BS, baseline condition; PSD, partial sleep deprivation; Rec = recovery condition.

These dynSampEn vectors were clustered into 5 distinct states for all subjects (Figure 4): state‐1 has the highest SampEn state, characterized by highly irregular spontaneous fluctuations across the whole brain (occurrence proportion of 13.81%); State‐2 has the second‐highest SampEn state, featuring relatively high entropy mainly in the temporal cortex and subcortical nuclei (occurrence proportion of 31.24%); State‐3 has an intermediate SampEn state with prominent subcortical but medium cortical entropy (occurrence proportion of 23.51%); State‐4 has the lowest SampEn state with globally suppressed flexibility (occurrence proportion of 13.36%); State‐5 was characterized by markedly weak SampEn within the occipital cortex (occurrence proportion of 18.09%). Brain activity across State‐1 through State‐5 would signify a change from strong complexity and flexibility (State‐1) to regularity and predictability (State‐4), with State‐5 representing a spatially specific pattern of constrained activity in occipital cortices.

**FIGURE 5:**
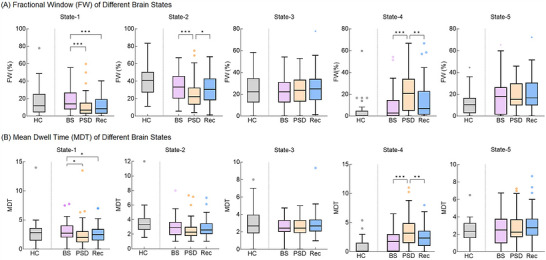
Dynamic features of different brain states. (A) Fractional window features of five brain states for healthy controls (HC), and night‐shift workers (NSW). (B) Mean dwell time features of five brain states for HCs and NSWs. BS, baseline condition; PSD, partial sleep deprivation; Rec = recovery condition. *, **, and *** represent *p* < 0.05, *p* < 0.01, and *p* < 0.001 after the Tukey's multiple comparison correction, respectively.

**FIGURE 6:**
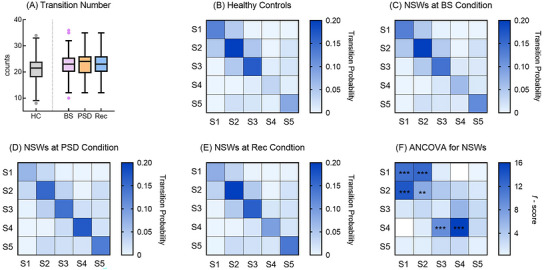
Dynamic brain state transition. (A) Total numbers of state transition during the rs‐fMRI scan. (B) Group‐averaged matrix of state transition probability for healthy controls (HC). (C–E) Group‐averaged matrix of state transition probability for night‐shift workers (NSW) at BS, PSD, and Rec conditions, respectively. (F) One‐way repeated measures ANCOVA analysis to estimate the effects of acute PSD on state transition probability. BS, baseline condition; PSD, partial sleep deprivation; Rec = recovery condition; *, **, and *** represent *p* < 0.05, *p* < 0.01, and *p* < 0.001 with FDR multiple correction, respectively.

One night of PSD led to a decrease in the occurrence (FW) and duration (MDT) for high SampEn State‐1 and State‐2, as well as an increase of these features for the lowest SampEn State‐4 (Figure 5). Interestingly, these dynamic features of the highest SampEn State‐1 remained significantly different from baseline condition even after 3–5 days of regular sleep, unlike the recovered State‐2 and State‐4. Furthermore, the acute PSD altered the state transition and persistence probabilities (one‐way repeated measures ANCOVA, *P* < 0.05, FDR correction), while placing no effect on the total number of state transitions during the rs‐fMRI scan (Figure 6 and Figure S7). Both the transition probability between the two high SampEn states (State‐1 and State‐2) and their persistence probabilities were significantly reduced after PSD compared to the baseline condition (*p* < 0.01, with Tukey's multiple comparison correction). In contrast, the persistence probability of the lowest SampEn State‐4 (*p* < 0.001, with Tukey's multiple comparison correction) and the transition probability between State‐4 and State‐3 were significantly elevated (*p* < 0.01, with Tukey's multiple comparison correction). Finally, the state transition probability returned to the baseline level following several days of sleep recovery, except for the transition probability between State‐1 and State‐2 and the persistence probability of State‐1 (*p* < 0.01, with Tukey's multiple comparison correction). In summary, the acute PSD shifted the temporal dynamics of brain activity away from high‐entropy, flexible conditions (States‐1 and 2) toward a low‐entropy, rigid state (State‐4), with the highest‐entropy state remaining disrupted even after sleep recovery.

There was no inter‐group difference in the dynamic temporal features between HCs and NSW participants at the baseline condition, or between the PSD‐Res and PSD‐Vul subgroups for any condition (all *p* > 0.05 with FDR correction).

### 3.4 PSD‐Res and PSD‐Vul by PVT Performance

According to the PVT performance of BS, PSD, and Rec conditions, 34 NSWs were categorized as PSD‐Res with both $\Delta_{PSD-BS}$ and $\Delta_{PSD-Rec}$ less than or equal to two in the PVT tests, while the other 35 NSW participants were categorized as PSD‐Vul with either $\Delta_{PSD-BS}>2$ or $\Delta_{PSD-Rec}>2$. There were no significant differences between these two subgroups in age, sex, BDI‐II, AIS, PSQI total and sub‐item scores, or the PVT meanRT at the BS and Rec conditions (Table 1). The NSW patterns were comparable between PSD‐Res and PSD‐Vul subgroups (Figure S8).

In addition, the inter‐condition differences in PVT performance were estimated among BS, PSD, and Rec conditions for PSD‐Res and PSD‐Vul subgroups, respectively (Figure S9). For the PSD‐Vul subgroup, both the meanRT and minor lapse counts after PSD were significantly increased compared to the BS and Rec conditions (all *p* < 0.01), whereas for PSD‐Res, no inter‐condition difference was identified in the meanRT (*p* = 0.09) or minor lapse counts (*p* = 0.33).

### 3.5 Shared and Subgroup‐Specific Alterations in Static SampEn Following PSD

**FIGURE 7:**
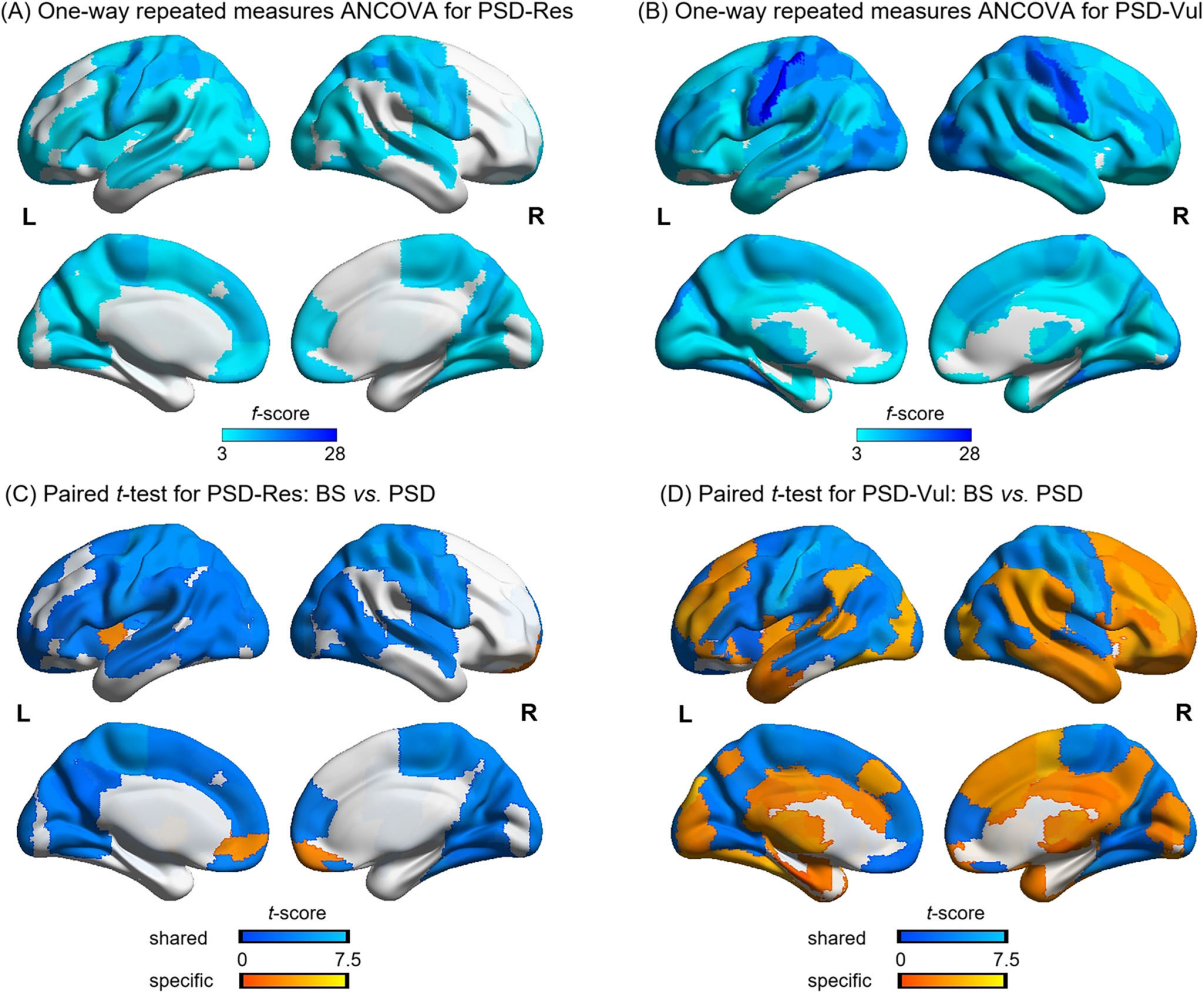
Shared and subgroup‐specific regions with reduced static SampEn following acute PSD. (A–B) The spatial distribution of sensitive brain regions for PSD‐Res and PSD‐Vul subgroups, respectively, where significant differences in static SampEn features were detected among BS, PSD, and Rec conditions (*p* < 0.05, with FDR correction). (C–D) The spatial distribution of brain regions for PSD‐Res and PSD‐Vul subgroups, respectively, where significant SampEn decline was detected following PSD, compared to BS condition (*p* < 0.05, with FDR correction). The cool color represents that the alteration pattern was shared by both PSD‐Res and PSD‐Vul subgroups, while the warm color represents that the alteration pattern was specific to either the PSD‐Res or the PSD‐Vul subgroup.

Compared to the baseline condition, the static SampEn following acute PSD significantly declined in distributed cortical regions for both the PSD‐Res and PSD‐Vul subgroups (*p* < 0.05, FDR correction), and largely recovered to the baseline level after 3–5 days’ regular sleep (*p* > 0.05, FDR correction) (Figure 7A,B). Altered SampEn in PSD‐Vul was more extensive in space as compared with the PSD‐Res subgroup (174 ROIs vs. 80 ROIs, Brainnetome atlas). The sensitive regions shared by these two PSD subgroups mainly involved the SMN, VN, and dorsal attention network (DAN), while the sensitive regions specific to PSD‐Vul mainly involved the anterior and middle parts of the default mode network (DMN), the occipital cortex within VN, the ventral attention network (VAN), the limbic network, the fronto‐parietal network (FPN), and the thalamus nuclei (Figure 7D). In summary, acute PSD induced widespread reductions in static SampEn, which were largely recovered after regular sleep, with PSD‐Vul individuals showing more extensive alterations involving higher‐order networks.

### 3.6 Classification of PSD‐Vul and PSD‐Res Individuals Based on SampEn

**FIGURE 8:**
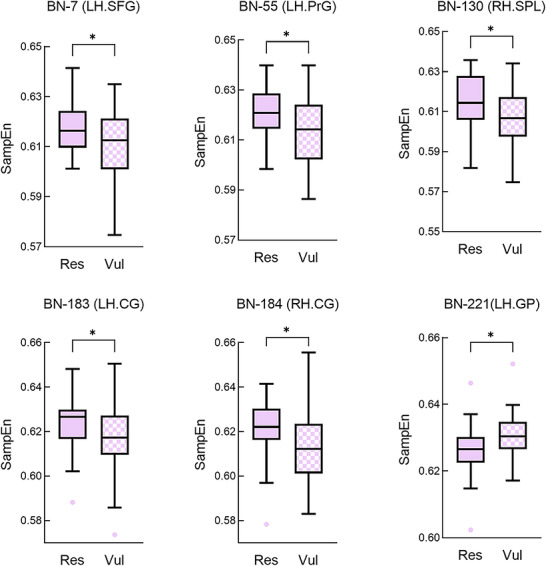
Brain regions with their SampEn metrics sensitive to individual vulnerability to partial sleep deprivation. PSD = partial sleep deprivation, Res = PSD‐resistant; Vul = PSD‐vulnerable; LH = left hemisphere, RH = right hemisphere, SFG = superior frontal gyrus, PrG = precentral gyrus, SPL = superior parietal lobule, CG = cingulate gyrus; GP = globus pallidus. * represents a significant statistical difference (*p* < 0.05) based on the two‐sample *t*‐test without multiple comparison correction.

The baseline SampEn metrics of Brainnetome ROIs were compared between PSD‐Res and PSD‐Vul night‐shift participants, with the age and head motion (mean FD) as covariances. The Res‐Vul difference of SampEn was detected in 6 ROIs, including the globus pallidus (GP, BN‐221) and areas belonging to the dorsal and ventral attention network (BN‐7, BN‐55, BN‐183, and BN‐184) (Figure 8). For these cortical ROIs, the SampEn features were significantly higher in the PSD‐Res subgroup relative to PSD‐Vuls, whereas the opposite pattern was found for the GP (*p* < 0.05, without FDR correction).

Subsequently, the L1‐regularization LR model was constructed based on these sensitive SampEn features. The optimal LR model achieved a classification accuracy of 78.26% (54/69) under the LOOCV, with an area under the curve (AUC) of 0.803, a sensitivity of 71.43%, and a specificity of 85.29%. A 1000‐time permutation test confirmed the statistical significance of the model (*p* = 0.001), suggesting the feasibility of SampEn in predicting the individual resistance to sleep deprivation. Collectively, these results suggest that baseline SampEn features may potentially help distinguish the PSD susceptibility at the individual level.

## 4 Discussion

In this study, we investigated the impacts of acute and chronic sleep deprivation on the complexity of spontaneous brain activity in a cohort of young female night‐shift medical staff based on resting‐state fMRI data and brain sample entropy analysis. Compared to HCs, this NSW cohort showed lower SampEn predominantly in the occipital and superior temporal cortices, suggesting declined flexibility in spontaneous brain activity linked to chronic occupational sleep deprivation. These NSW participants were further categorized into PSD resistant and vulnerable subgroups according to their PVT performance. After the acute PSD in a night‐shift work, an extensive decline in brain entropy was detected for NSWs with shared and subgroup‐specific patterns. The alterations in both static and major dynamic SampEn features recovered to baseline following several days of regular sleep. The baseline SampEn features combined with an L1‐regularized logistic regression model enabled an effective discrimination of PSD‐Res and PSD‐Vul individuals, advancing our understanding of the linkage between local brain activity and individual differences in psychomotor vigilance after sleep deprivation.

### 4.1 Declined Complexity of Spontaneous Brain Activity Following Acute PSD

Acute PSD led to a prevailing decline in static brain entropy and a shift of temporal dynamics from the predominance of high‐entropy states to the persistence of low‐entropy states, indicating that the spontaneous fluctuation of brain activity was organized towards a more regular paradigm with constrained neural flexibility. This finding was consistent with previous studies reporting the flexibility loss of resting‐state brain activity after total sleep deprivation characterized by lower‐dimensional and restricted energy landscapes (Wu et al. 2025) and decreased wavelet packet entropy (Wang et al. 2019). This rigid temporal organization pattern may restrict the responsiveness of the brain to the intrinsic or external stimuli, thereby impairing the psychomotor vigilance and neurocognitive performance following sleep deprivation. A recent study proposed that the altered brain dynamics after sleep deprivation may arise from the growing energy requirement modulated by the redistributed striatal dopamine D2/D3 receptors (R. Zhang et al. 2025). Collectively, the declined complexity of the brain activity in the current PSD cohort suggests an imbalance in functional integration and segregation that is potentially substrated by aberrant molecular mechanisms (Volkow et al. 2009; Tomasi et al. 2016).

Nevertheless, the PVT performance and the altered SampEn were both reversed after several days of regular sleep, highlighting the remarkable plasticity ability of the human brain in response to acute PSD (Kalkanis et al. 2023). In contrast, the alterations of functional connectivity following sleep deprivation seem to be more complicated and prolonged (Yao et al. 2025), suggesting the vulnerability of inter‐regional information dissemination compared to local neural oscillation. Future work that combines the analyses of brain entropy and connectivity analysis may contribute to elucidating the coordinated or distinct regulation patterns of the local brain activity and global arrangement in response to accumulative sleep pressure, thus furthering our understanding of the effects of sleep deprivation on brain function.

### 4.2 Neural Correlates of Individual Difference in Psychomotor Vigilance After Acute PSD

Acute and chronic sleep restriction leads to mental fatigue and neurocognitive impairment with individual differences. Emerging evidence indicates that the individual different response is stable and trait‐like (Yamazaki and Goel 2020), emphasizing the presence of underlying intrinsic neurobiological and genetic substrates (Satterfield et al. 2015; Holst et al. 2017). We found a widespread decline in the brain entropy after one‐night PSD for both PSD‐Res and PSD‐Vul participants, with more widespread brain regions identified for the PSD‐Vul subgroup, suggesting the susceptibility of their neural complexity modulation to sleep deprivation. The additional involvement in the brains of PSD‐Vul was primarily located in the frontal and temporal cortices and the thalami. Since the thalamus plays a key role in high‐level cognition, visuospatial perception, and attention (Bourbon‐Teles et al. 2014), the specific alteration pattern of the thalamus may partially underpin the worsened sustained attention and alertness in PSD‐Vul subjects as compared to the PSD‐Res individuals.

In addition, the neural complexity under the baseline wakefulness was different between NSW subgroups. Compared to PSD‐Vul, PSD‐Res showed higher SampEn in brain regions belonging to dorsal and ventral attention networks, signifying a highly flexible state of the spontaneous fluctuation in the neural ensembles of these higher‐order networks. The baseline level of brain local activation and functional connectivity may lay the foundation of individual resilience to sleep deprivation (Mu et al. 2005; Yeo et al. 2015). Combined with the logistic regression model, the sensitive SampEn features achieved a high accuracy in discriminating PSD‐Res and PSD‐Vul individuals at the baseline condition, indicating the feasibility of brain entropy as a novel biomarker of the individual's susceptibility to sleep deprivation. By capturing the temporal irregularity of local spontaneous brain activity, BEN offers a complementary perspective on the neural basis underlying inter‐individual differences in vulnerability to partial sleep restriction. Future work incorporating BEN with other neural correlates, such as functional connectivity and white matter diffusion metrics (Yeo et al. 2015; C. Wang et al. 2022) in a multimodal framework with larger cohorts may further improve the identification of PSD susceptibility.

It should be noted that the behavioral phenotyping relied solely on PVT minor lapse counts in the present study. Despite the high reproducibility of PVT, this threshold‐based stratification may constrain the generalizability of our subgroup‐specific findings (Yamazaki et al. 2022). Future studies incorporating more robust stratification criteria (e.g., genetic biomarkers) alongside continuous correlation analyses between BEN and PVT performance would promote the elucidation of the quantitative relationship between neural complexity and individual vulnerability to sleep deprivation.

### 4.3 Potential Effects of Long‐Term Night‐Shifts on Local Neural Complexity

Reduced irregularity of the brain activity, particularly within the occipital and temporal cortices that belong to SMN and VN functional networks, was found to be correlated with the poorer sleep quality of the night‐shift subjects in this study, highlighting the preferential susceptibility of primary functional networks to chronic PSD. Despite the classically recognized involvement in primary visual information processing and visual attention, the occipital cortex was recently reported to play an important role in the regulation of sleep state transition characterized by elevated activation during the rapid‐eye‐movement (REM) sleep state (Z. Wang et al. 2022). The functional activity of the occipital and temporal cortices was found to be associated with the impairment of salience detection and sustained attention following long‐term or one‐night of night‐shift work (Kim et al. 2023; Peng et al. 2024). Given that the disturbance of the brain complexity modulation is typically identified in various psychiatric and neurodegenerative disorders (Zheng et al. 2020; Kim et al. 2024), the observed reduction of the neural complexity in the occipital and temporal cortices may signify a convergent mechanism underlying the cognitive impairments in female NSW subjects with long‐term PSD exposure.

However, these nominal changes relative to HCs did not survive the correction for multiple testing, which may be partly attributed to the relatively young age of the PSD participants of this study. Emerging evidence suggests that the neurobiological consequences of sleep deprivation are age‐dependent, with younger brains exhibiting more flexible compensatory network configurations in response to sleep loss (Wu et al. 2026). Furthermore, the relatively short duration of occupational night‐shift exposure in our cohort may be insufficient to lead to pronounced alterations in neural complexity, given that shift‐work‐associated cognitive impairments tend to accumulate with prolonged exposure (Marquié et al. 2015). Future studies involving older subjects with extended shift‐work histories would help elucidate the trajectory and cumulative impact of prolonged night‐shift work on brain function.

### 4.4 Limitations

There were several limitations of this study. First, the sleep structure data was not available in this study. The self‐report PSQI and AIS questionnaires were utilized for the estimation of sleep quality. This may limit the investigation of specific effects of PSD on different sleep stages (Marzano et al. 2010; Yook et al. 2024). Future work incorporating objective sleep structures may provide additional correlates of night‐shift work to sleep homeostasis. Second, the timing, duration, and pattern of napping were not objectively recorded at the time of data collection. Napping during NSW may mitigate the PSD‐induced impairments of brain function (X. Zhang et al. 2025), confounding the impacts of PSD on the complexity of brain activity in the current study. Third, the female NSW participants of the present study were relatively young and junior. Current findings may not be sufficient to capture the PSD impacts for populations of different sex and age groups.

## 5 Conclusions

The temporal irregularity of spontaneous brain fluctuations is sensitive to acute PSD and demonstrates reversibility following recovery sleep. Chronic PSD associated with night‐shifts leads to a trend of local flexibility reduction, bringing new insights into the neural substrates driving sleep disturbances in female medical staff with long‐term PSD exposure. Brain entropy metrics characterizing neural complexity not only further our understanding of PSD impacts on brain function, but also provide a potential biomarker for predicting the individual's susceptibility to sleep deprivation, which would assist the optimization of rotating‐shift schedules.

## Author Contributions

**Lixian Zou**: methodology, writing – review and editing, funding acquisition. **Fan Yang**: writing – review and editing, formal analysis, data curation, methodology. **Lijuan Zhang**: conceptualization, investigation, funding acquisition, writing – original draft, writing – review and editing, methodology, formal analysis, project administration, data curation, supervision, resources. **Siqi Cai**: investigation, funding acquisitio, writing – original draft, writing – review and editing, methodology, formal analysis, data curation. **Chunxiang Jiang**: investigation, funding acquisition, writing – review and editing, writing – original draft.

## Funding

This work was partially supported by National Natural Science Foundation of China (82341248, 82302177), Strategic Priority Research Program of the Chinese Academy of Sciences (XDB0930000), Shenzhen Science and Technology Program (GJHZ20220913142812024), Guangdong Basic and Applied Basic Research Foundation (2021A1515010200), Key Laboratory of Biomedical Imaging Science and System, Chinese Academy of Sciences, and State Key Laboratory of Biomedical Imaging Science and System.

## Conflicts of Interest

All authors declare that they have no biomedical financial interests or potential conflicts of interest.

## Supporting information




**Supplementary Material**: brb371530‐sup‐0001‐SuppMat.pdf

## Data Availability

The sharing of raw MRI data was prohibited according to the research ethics regulated by the local institutional review board. Anonymous questionnaires about subjective sleep quality, data from PVT experiment and brain entropy analysis, and custom‐written Matlab scripts are available via a request to the corresponding author.
