# Supplementary material for: Reduced but Reversible Brain Entropy After Occupational Partial Sleep Deprivation in Night‐Shift Medical Staff
Source: Brain Behav. 2026 Jun 19;16(6):e71530. doi: 10.1002/brb3.71530 (PMC13280564; doi:10.1002/brb3.71530)

Supplementary Material

Table S1 Sub-item scores of PSQI Questionnaire

Sub-item	NSWs (n = 69)	HCs (n = 30)	Statistical Analysis		PSD-Res (n = 34)	PSD-Vul (n = 35)	Statistical Analysis	
			Z/t	***P***			***Z/t***	***P***
**Subjective Sleep Quality**	1 [0, 3]	1 [0, 2]	-2.52	0.01*	1 [0, 2]	1 [0, 3]	-0.25	0.80
**Sleep Latency**	2[0, 3]	1 [0, 2]	-3.38	<0.001*	2 [0, 3]	1 [0, 3]	-0.89	0.37
**Sleep Duration (hours)#**	6.31 ± 1.12	7.60 ± 0.90	-5.58	<0.001*	6.41 ± 1.22	6.21 ± 1.02	0.78	0.44
**Sleep Duration (score)**	1 [0, 3]	0 [0, 1]	-4.96	<0.001*	1 [0, 3]	1 [0, 3]	-0.74	0.46
**Sleep Efficiency (%)#**	87.45 ± 11.53	91.94 ± 7.28	-1.97	0.05	87.62 ± 10.18	87.28 ± 12.86	0.12	0.90
**Sleep Efficiency (score)**	0 [0, 3]	0 [0, 1]	-1.90	0.06	0 [0, 3]	0 [0, 3]	-0.79	0.43
**Sleep Disturbance**	1 [0, 2]	1 [0, 1]	-2.12	0.03*	1 [0, 2]	1 [0, 2]	-0.28	0.78
**Daytime Dysfunction**	2 [0, 3]	1 [0, 2]	-3.74	<0.001*	2 [0, 3]	2 [0, 3]	-1.07	0.28

Notes: # represents the two-sample t-test (two-tailed) with data expressed as mean ± standard deviation, while the other data were expressed as median [minimum, maximum] and estimated with Mann-Whitney U test.

Figure S1. Optimal parameters for static sample entropy (SampEn) analysis. According to the published strategy of parameter selection (Xin et al., Front Neurosci. 2024;18:1352409; Yang et al., Front Neurosci. 2018;12:398.), the CSF BOLD fMRI signals of healthy and night-shift participants were extracted and utilized as reference signals. Then, the relative error of SampEn of CSF signals was calculated in a range of *m* (1, 2, 3) and *r* (0.20 to 0.60 in the step of 0.02). The optimal parameters were set as *m* = 2 and *r* = 0.24 in this study, which correspond to the minimal relative error of SampEn for CSF signals.

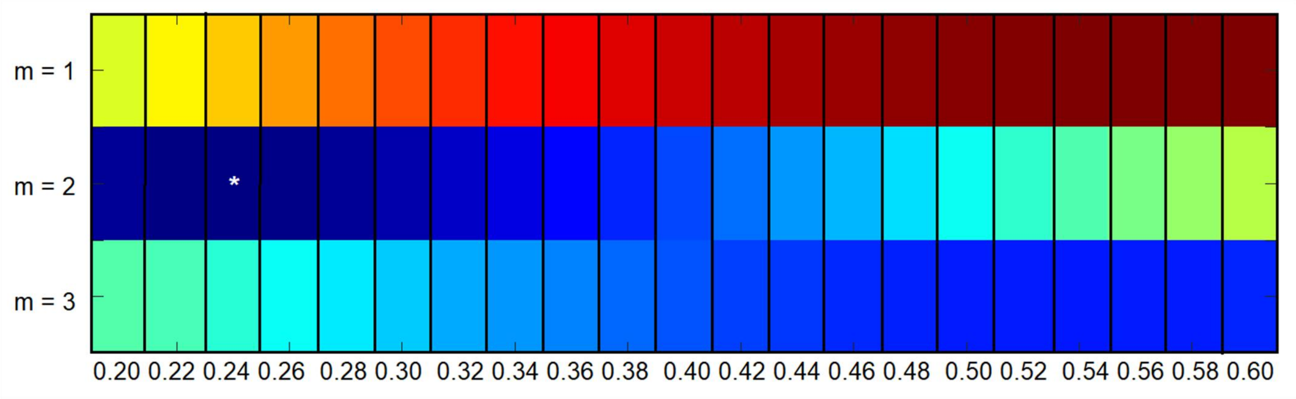

Figure S2. Optimal Cluster Number Selection for *K*-means Analysis. For each value of *K* ranging from 2 to 20, clustering analyses were conducted ten times on randomly selected 20% samples. The optimal cluster number was identified as *K* = 5, because the additional variance explained by increased *K* beyond *K* = 5 fell below 1%.

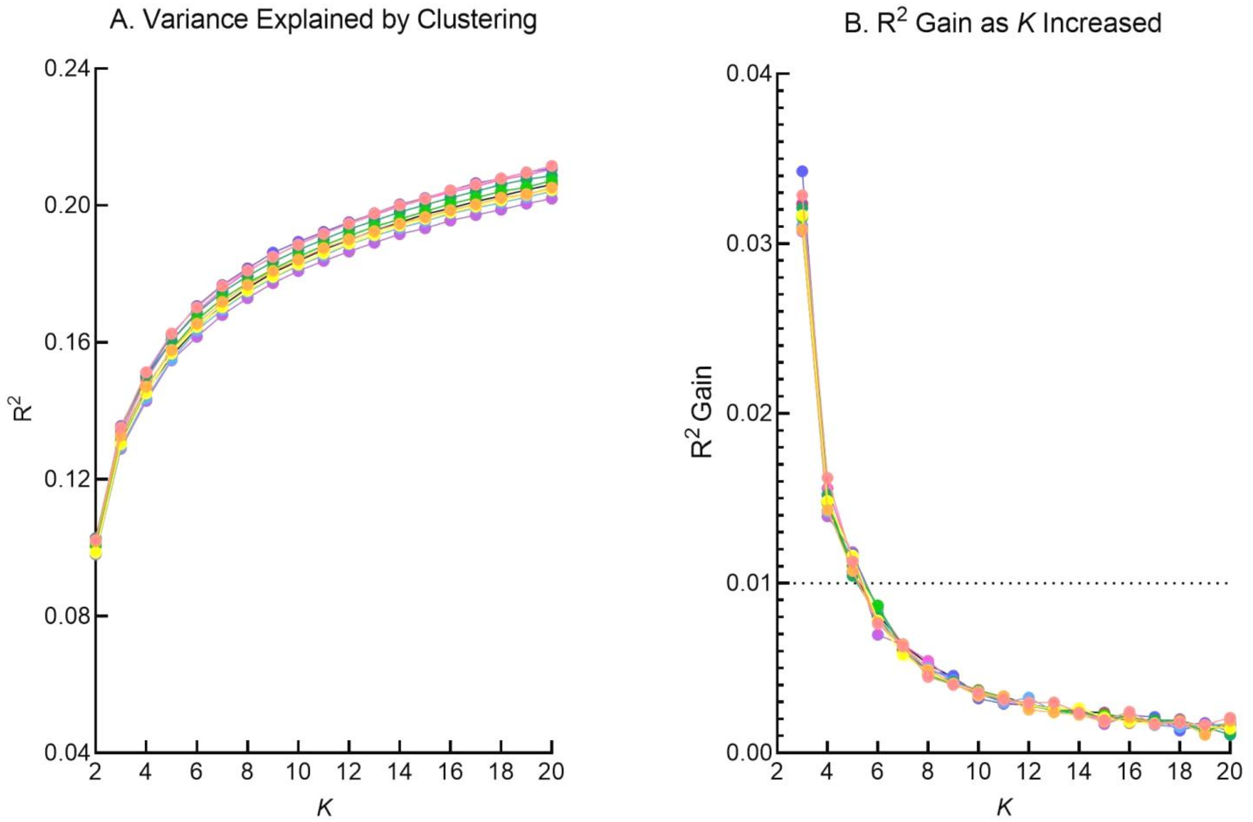

Figure S3. Illustration of the workflow for the individual classification of PSD-resistant and PSD-vulnerable subjects. A leave-one-out cross-validation (LOOCV) framework was applied to separate the night-shift participants into train and test datasets. In each LOOCV iteration, the principal component analysis (PCA) was applied to the training set using candidate SampEn features that showed inter-subgroup difference (PSD-Vul vs. PSD-Res, Two-sample *t*-test, $P < 0.05$). The first components accounting for 85% of the cumulative variance were retained as predictive features. The L1-regularized logistic regression (LR) model was then trained on the dimension-reduced training set, with the regularization parameter (λ) optimized via inner 5-fold cross-validation. The optimized model was subsequently used to predict the held-out test sample.

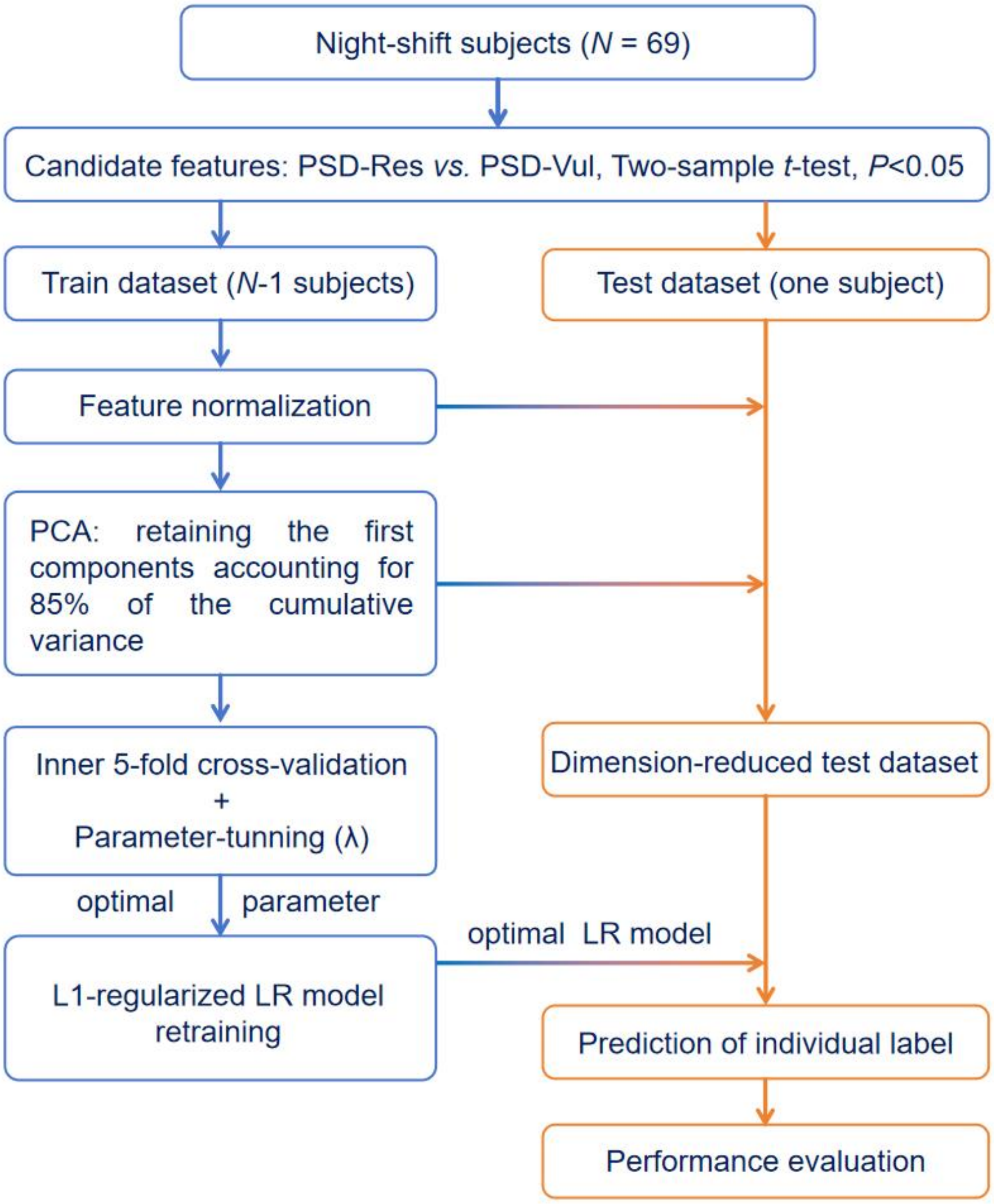

Figure S4. Head motion parameters of participants during the resting-state fMRI scan. Two-sample *t*-test analysis was performed to assess the difference in mean framewise displacement (FD) between healthy controls (HC) and night-shift workers (NSW) with no inter-group difference being identified. One-way repeated measure ANOVA was performed to assess the difference in mean FD among three conditions for NSWs, followed by Tukey's multiple comparison test for *post hoc* pairwise comparison. The head motion of PSD condition was significantly larger than that of baseline condition. BS = baseline condition before a night-shift work, PSD = partial sleep deprivation condition following a night-shift work, Rec = recovery condition with 3-5 days of regular sleep after PSD.

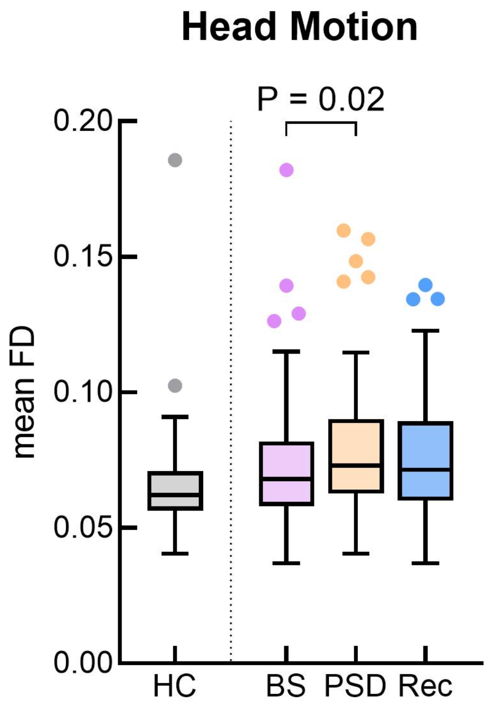

Figure S5. Group-averaged SampEn maps for healthy controls (HC) and night-shift participants (NSW) at the baseline (BS), partial sleep deprivation (PSD), and recovery (Rec) conditions. The SampEn metrics of most brain regions significantly decreased following one-night PSD, and largely recovered to the baseline level after several days' regular sleep.

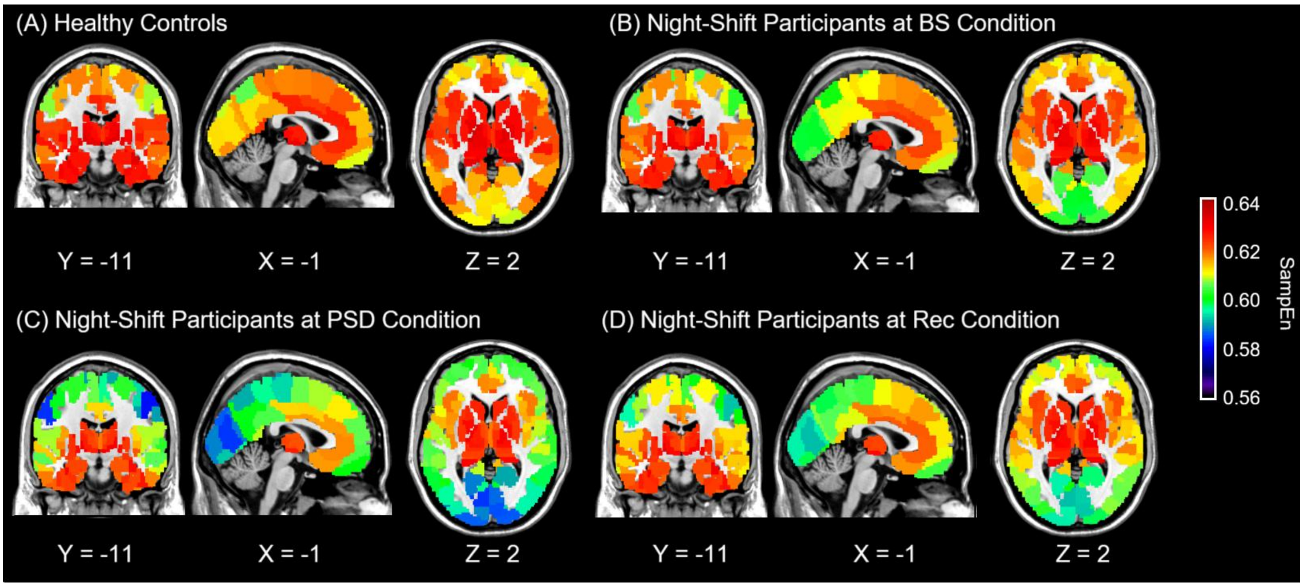

Figure S6. Sensitive brain regions with lower static sample entropy (SampEn) in group of night-shift participants (NSW), as compared with healthy controls (HC). (Two-sample *t*-test, *P* <0.05, uncorrected). L = left hemisphere, R= right hemisphere, OrG = orbital gyrus, PCL = paracentral lobule, PCun = precuneus, PoG = postcentral gyrus, INS = insular gyrus, CG = cingulate gyrus, STG = superior temporal gyrus, FuG = fusiform gyrus, PhG = parahippocampal gyrus, MVOcC = medioventral occipital cortex, LOcC = lateral occipital cortex, Amyg = amygdala, NAC = nucleus accumbens, Tha = thalamus. The gray and pink boxes represent groups of HCs and NSWs, respectively.

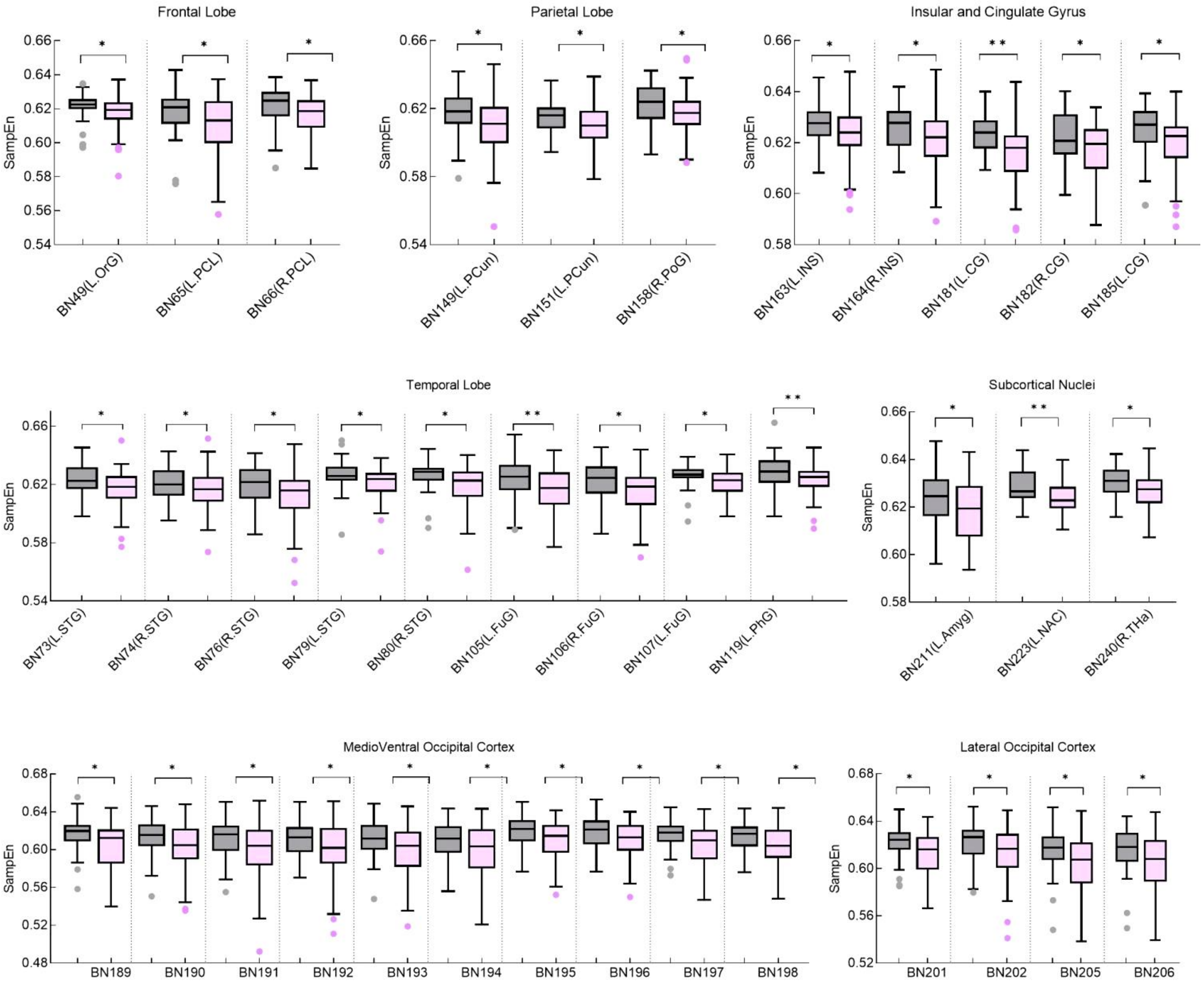

Figure S7. One-way repeated measure ANCOVA analysis to estimate the effects of acute PSD on state transition probability. BS, baseline condition; PSD, partial sleep deprivation; Rec = recovery condition; *, **, and *** represent $P < 0.05$, $P < 0.01$, and $P < 0.001$ after Tukey's multiple comparison correction, respectively.

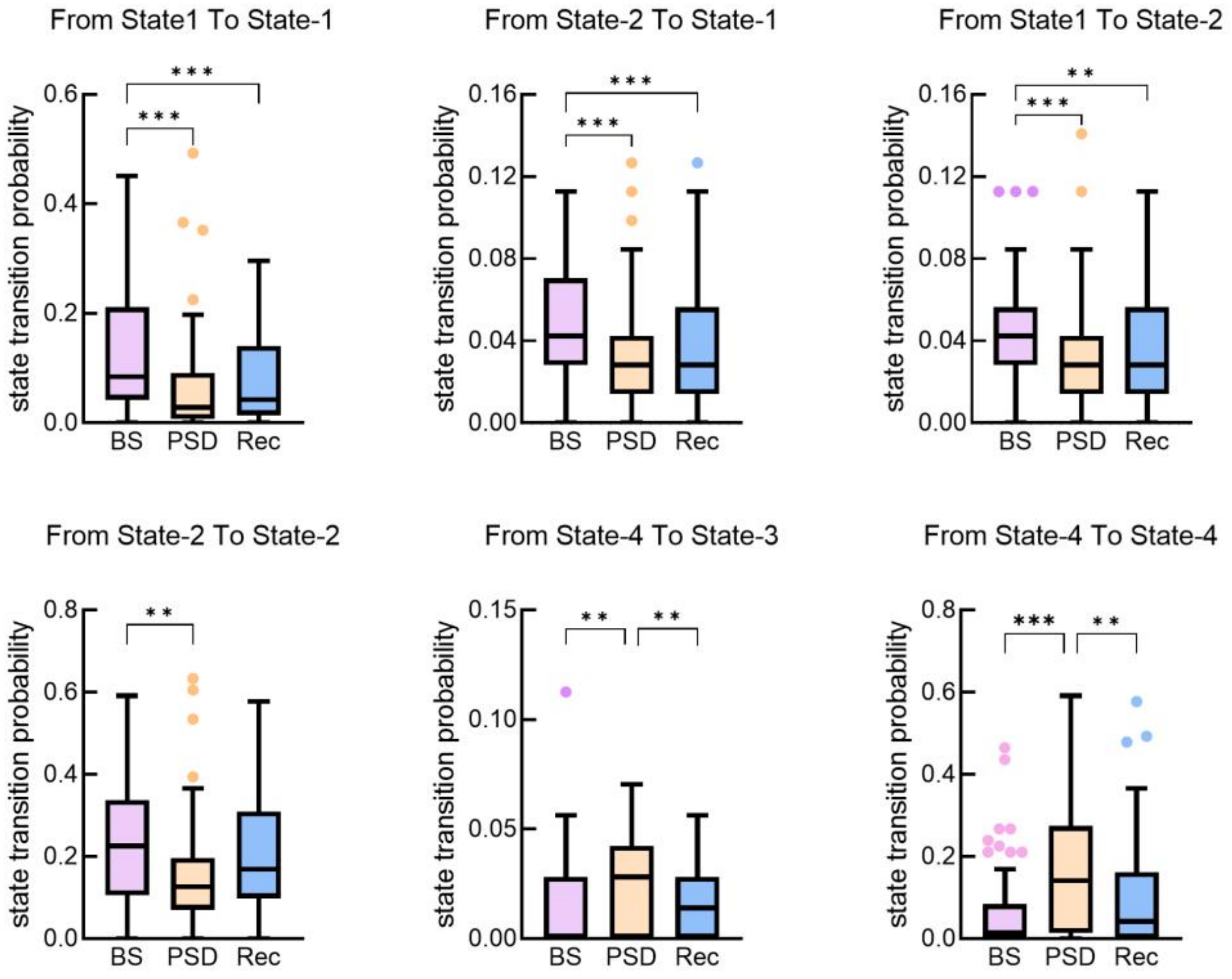

Figure S8. Night-shift patterns of PSD-resistant (Res) and vulnerable (Vul) night-shift participants. A Chi-square test was utilized to estimate the night-shift indices between subgroups of PSD-Res and PSD-Vul night-shift participants, with no inter-group difference being detected (*P* > 0.05).

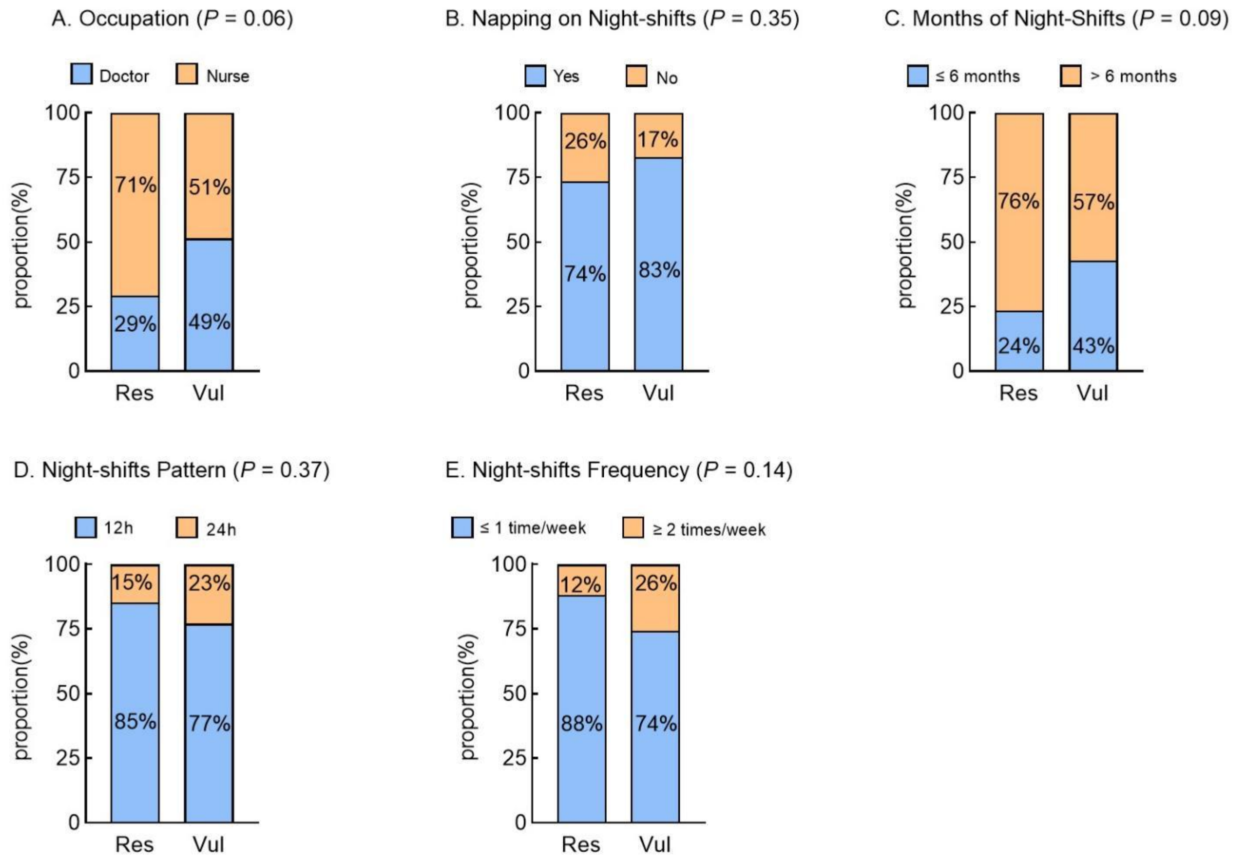

Figure S9. Psychomotor vigilance (PVT) performance of PSD-resistant and vulnerable night-shift participants at three conditions. One-way repeated measures ANOVA was performed to assess the difference in mean reaction time (meanRT) among three conditions, followed by Tukey's multiple comparison for *post hoc* pairwise comparison. Friedman non-parametric repeated measures ANOVA was performed to assess the difference in minor lapse counts among three conditions, followed by Dunn's multiple comparison test. For the PSD-vulnerable subgroup, both the meanRT and minor lapse counts after PSD were significantly increased compared to the BS and Rec conditions (all $P < 0.001$), whereas for PSD-Res, no inter-condition difference was identified. BS = baseline condition before a night-shift work, PSD = partial sleep deprivation condition following a night-shift work, Rec = recovery condition with 3-5 days of regular sleep after PSD. *, ** and *** represent $P < 0.05$, $P < 0.01$ and $P < 0.001$, respectively.

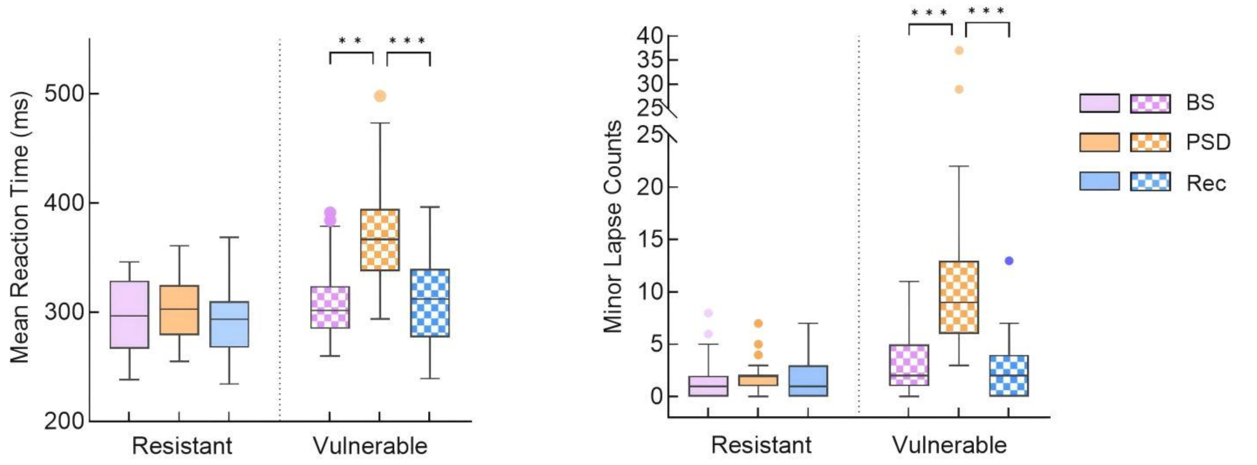

Supplement: Supplementary file 1 — Supplementary Material: brb371530‐sup‐0001‐SuppMat.pdf [file BRB3-16-e71530-s001.pdf]
